# Intestinal microbiota and metabolome perturbations in ischemic and idiopathic dilated cardiomyopathy

**DOI:** 10.1186/s12967-023-04605-6

**Published:** 2024-01-22

**Authors:** Yusheng Wang, Yandan Xie, Gehendra Mahara, Yanling Xiong, Yalan Xiong, Qifang Zheng, Jianqin Chen, Wei Zhang, Honghao Zhou, Qing Li

**Affiliations:** 1 Department of Cardiovascular Internal Medicine, The First Affiliated Hospital of Shantou University Medical College, Shantou, 515041 Guangdong China; 2 Clinical Research Center, Shantou University Medical College, Shantou, 515041 Guangdong China; 3 Department of Clinical Pharmacology, Xiangya Hospital, Central South University, Changsha, 410008 Hunan China

**Keywords:** Idiopathic dilated cardiomyopathy, Ischemic dilated cardiomyopathy, Microbiota, Metabolome, Multi-omics analysis

## Abstract

**Background:**

Various clinical similarities are present in ischemic (ICM) and idiopathic dilated cardiomyopathy (IDCM), leading to ambiguity on some occasions. Previous studies have reported that intestinal microbiota appeared dysbiosis in ICM, whether implicating in the IDCM remains unclear. The aim of this study was to assess the alterations in intestinal microbiota and fecal metabolites in ICM and IDCM.

**Methods:**

ICM (n = 20), IDCM (n = 22), and healthy controls (HC, n = 20) were enrolled in this study. Stool samples were collected for 16S rRNA gene sequencing and gas chromatography-mass spectrometry (GC–MS) analysis.

**Results:**

Both ICM and IDCM exhibited reduced alpha diversity and altered microbial community structure compared to HC. At the genus level, nine taxa including *Blautia, [Ruminococcus]_torques_group, Christensenellaceae_R-7_group, UCG-002, Corynebacterium, Oceanobacillus, Gracilibacillus, Klebsiella* and *Citrobacter* was specific to ICM, whereas one taxa *Alistipes* uniquely altered in IDCM. Likewise, these changes were accompanied by significant metabolic differences. Further differential analysis displayed that 18 and 14 specific metabolites uniquely changed in ICM and IDCM, respectively. The heatmap was generated to display the association between genera and metabolites. Receiver operating characteristic curve (ROC) analysis confirmed the predictive value of the distinct microbial-metabolite features in disease status. The results showed that microbial (area under curve, AUC = 0.95) and metabolic signatures (AUC = 0.84) were effective in discriminating ICM from HC. Based on the specific microbial and metabolic features, the patients with IDCM could be separated from HC with an AUC of 0.80 and 0.87, respectively. Furthermore, the gut microbial genus (AUC = 0.88) and metabolite model (AUC = 0.89) were comparable in predicting IDCM from ICM. Especially, the combination of fecal microbial-metabolic features improved the ability to differentiate IDCM from ICM with an AUC of 0.96.

**Conclusion:**

Our findings highlighted the alterations of gut microbiota and metabolites in different types of cardiomyopathies, providing insights into the pathophysiological mechanisms of myocardial diseases. Moreover, multi-omics analysis of fecal samples holds promise as a non-invasive tool for distinguishing disease status.

**Graphical Abstract:**

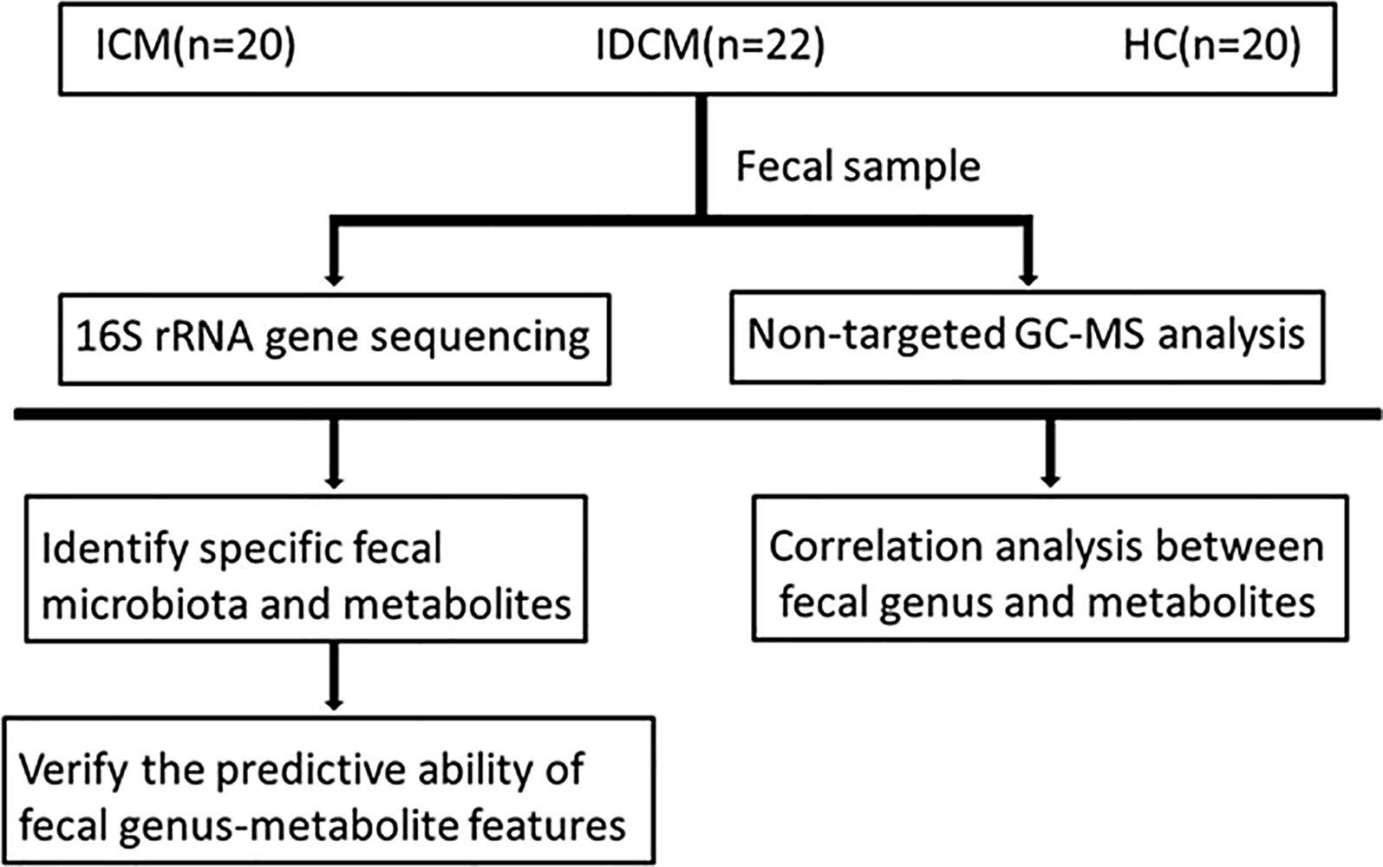

**Supplementary Information:**

The online version contains supplementary material available at 10.1186/s12967-023-04605-6.

## Introduction

Dilated cardiomyopathy (DCM) encompasses a diverse group of cardiac disorders characterized by structural or functional abnormalities of the heart, often leading to heart failure [1, 2]. Ischemic dilated cardiomyopathy (ICM) occurs when severe coronary artery disease impedes blood flow to the energy-dependent cardiomyocytes, resulting in pump dysfunction [3, 4]. On the other hand, idiopathic dilated cardiomyopathy (IDCM) is recognized as another common cause of heart failure in the absence of coronary artery disease, hypertension or valvular heart disease. Previous studies have revealed several etiologies involved in the process of IDCM, including genetic variation, infections, immunological abnormalities, and toxic effects of alcohol or drugs [5–8]. Differences in the underlying pathophysiology significantly influence the clinical characteristics, long-term outcomes, and management strategies for patients with cardiomyopathies [9–12]. Therefore, elucidating the etiology of cardiomyopathies holds the potential to develop personalized therapies and improve clinical outcomes. Currently, invasive techniques such as coronary artery angiography, as well as non-invasive image modalities like cardiac magnetic resonance imaging, and single-photon emission computed tomography, are used to distinguish ICM from IDCM [4, 13]. However, the associated risks and costs of these approaches remain a concern in clinical practice.

The human intestinal microbiota is a complex symbiotic ecosystem that plays an important role in the physiological state of the host. A growing body of evidence suggests the involvement of gut microbiota in various diseases [14–16]. Notably, patients with ICM exhibit distinct taxonomic and functional characteristics of the intestinal microbiota compared to healthy individuals. ICM is characterized by a reduction in bacterial abundance, such as *Kinetoplastids*, *Turcimonas*, and *Acetobacter*, along with an increased *Proteobacteria* [17]. Previous studies have also shown a significant association between the increased abundance of *Proteobacteria* and the severity of coronary lesions [18]. Conversely, the relationship between intestinal microbiota and IDCM remains unexplored. Dysbiosis of the gut microbiota can lead to physiological dysfunction, including disturbances in energy metabolism, synthesis of metabolites, pathogen resistance, and immune regulation. Whether dysbiosis in intestinal microbiota is related to the pathophysiological mechanisms of IDCM requires further investigation.

Natural metabolites derived from the gut microbiota have been found to exert multiple biological activities and play a vital role in the cross-talk between the microbiota and the host. Fecal metabolomics has the potential to shape the composition of the microbiota and regulate the metabolic homeostasis of the host [19, 20]. Furthermore, it has been suggested that fecal metabolomics can reflect gut microbial composition and explain a significant proportion of the variance in host metabolomics data [21]. Emerging evidence highlights the association between imbalances in metabolite profiles and cardiovascular diseases such as hypertension, heart failure, and cardiomyopathy [18, 22, 23]. Thus, integrated analysis of gut microbiota and metabolomics data to identify key features may provide valuable insights into disease processes.

To investigate the distinctive features of intestinal microbiota and fecal metabolites in patients with different phenotypes of cardiomyopathies, we performed 16S rRNA gene sequencing and GC–MS analysis on fecal samples obtained from a clinical cohort comprising 20 patients with ICM, 22 patients with IDCM, and 20 healthy controls (HCs). Based on the multi-omic data analysis, we sought to construct a disease classifier capable of differentiating IDCM from ICM.

## Materials and methods

### Study design and ethical approval

This is a cross-sectional study, aimed to investigate the alterations in gut microbiota and metabolites in different types of cardiomyopathies, specifically ischemic cardiomyopathy (ICM) and idiopathic dilated cardiomyopathy (IDCM). The study was conducted at the First Affiliated Hospital of Shantou University Medical College between February 2022 and April 2023 (ChiCTR2300074280).

#### Participants

Newly diagnosed patients with DCM were included in the study. The inclusion criteria were: (1) Left ventricular end‐diastolic diameter (LVEDd) > 5.0 cm for women or LVEDd > 5.5 cm for men; (2) Left ventricular ejection fraction (LVEF) < 45% according to Teichholtz method.

All participants underwent percutaneous coronary angiography to assess their coronary anatomy. The distinction between ICM and IDCM was based on the severity of coronary artery disease observed in coronary angiography. In particular, the clinical diagnosis of IDCM was based on unexplained ventricular enlargement and reduced myocardial systolic function in the absence of coronary artery disease. ICM was defined as left ventricular dilatation and systolic dysfunction accompanied by severe coronary artery disease (50% or greater diameter stenosis in at least one major coronary artery). Based on the results of echocardiography and coronary angiography, patients were categorized into two groups (ICM and IDCM) for statistical analysis. The exclusion criteria were as follows: (1) autoimmune diseases, including rheumatoid arthritis, systemic lupus erythematous, and hyperthyroidism; (2) history of digestive system diseases, including chronic hepatitis, cholecystitis, and pancreatitis; (3) irritable bowel syndrome or inflammatory bowel diseases such as Crohn’s disease and ulcerative colitis; (4) a history of cancer and gastrointestinal surgery; (5) acute and chronic infectious diseases; (6) severe primary valvular heart disease and uncontrolled hypertension contributing to ventricular dilatation; (7) congenital heart disease, restrictive cardiomyopathy, and tachycardiainduced cardiomyopathy; (8) alcoholism; (9) usage of antibiotic, probiotics and prebiotics within 3 months; (10) difficult to classify as ICM or IDCM according to coronary stenosis. Therefore, a total of 20 patients with ICM and 22 patients with IDCM were enrolled in this study. In addition, 20 HCs with no history of diseases or oral medication were included.

#### Ethical approval

The study was conducted in accordance with the Declaration of Helsinki and approved by the Ethics Committee of the First Affiliate Hospital Shantou University Medical College, China (Approval No. B-2022-288).

### Data collection

Clinical variables, laboratory data, medical history, medication history, and echocardiographic parameters were collected from electronic medical records. Left ventricular end-diastolic dimension (LVEDd), left ventricular end-systolic dimension (LVEDs), and left ventricular ejection fraction (LVEF) were calculated using the Teichholtz method. Coronary angiography and the Gensini score were used to access coronary artery lesions. Regional wall motion abnormality (RWMA) encompassed hypokinesis, akinesis, dyskinesis, and aneurysm [24]. The scoring system assigned a severity score to each coronary branch based on the degree and geographical importance of stenosis [25].

### Fecal sample collection, DNA extraction, and 16S rRNA gene sequencing

Fresh faecal samples were collected from patients with DCM and HC, transported to the laboratory on ice in a foam box, and stored at −80 °C until analysis. Total genomic DNA was extracted from the human fecal samples according to the cetyltrimethylammonium bromide and sodium dodecyl sulfate (CTAB-SDS) method. For alpha and beta diversity analysis, the V3-V4 region of the bacterial 16S rRNA gene was amplified using a specific primer with the barcode. All PCR reactions were performed using Phusion^®^ High-Fidelity PCR Master Mix (New England Biolabs). The PCR products were mixed in equal proportions and purified using the Qiagen Gel Extraction Kit (Qiagen, Germany). After these procedures, sequencing libraries were generated using the TruSeq^®^ DNA Free PCR Sample Preparation Kit (Illumina, USA), and index sequences were added. Finally, the libraries were sequenced on an Illumina NovaSeq 6000 platform (Illumina Inc., CA, USA) and 250 bp paired-end reads were generated.

### Analysis process of 16S rRNA gene sequencing data

After splicing, detecting, and removing chimeric sequences from raw sequencing data, effective tags were obtained. Using the Uparse algorithm (Uparse v7.0.1001, http://www.drive5.com/uparse/) [26], effective tags of all samples with an identity of 97% were clustered into operational taxonomic units (OTUs). The phylogeny of all OTUs representative sequences was obtained by MUSCLE software for rapid multiple sequence alignment [27].QIIME software (Version 1.9.1) was used to investigate diversity and differences in microbial community composition. Alpha diversity analysis was performed to assess the community richness and diversity using the Shannon, Simpson, and ACE indices. Beta diversity was evaluated by principal coordinate analysis (PCoA) based on weighted-unifrac distances to describe the microbial community composition among different groups. Metastats analysis was used to assess differences in the relative abundance of bacteria between the disease and control groups. MaAsLin analysis was performed to evaluate the effect of confounding factors on bacteria in different clinical groups.

### Sample preparation and GC–MS analysis

A total of 62 feces samples were categorized into three groups according to clinical types. Internal standards, L-norleucine and L-norvaline were purchased from Sigma-Aldrich (Shanghai, China). For the sample preparation, 80 μL of cold methanol with 5 μg/mL L-norleucine was added to extract metabolites from 20 ul of the supernatant obtained from a mixture of 100 mg feces and 100 ul water. After centrifuging at 4 °C and 14,000 × g for 15 min, 40 μL supernatants and 5 μL L-norvaline (50 μg/mL) were evaporated under a nitrogen stream. The dried products were reconstituted in 35 μL methoxyamine hydrochloride in pyridine (containing 5 μg/mL n-alkanes standards) (37 ℃, 90 min) and derivatized in 35 μL of BSTFA (with 1% TMCS) (70 ℃, 60 min) for GC–MS metabolomics analysis. Additionally, 100 µL of each fecal sample was mixed as a quality control (QC) sample.

### GC–MS data acquisition

Non-targeted metabolomics analysis of the fecal samples was performed using gas chromatography–mass spectrometry (7890A/5975C GC–MS System, Agilent, CA, USA). The derivatives were separated in OPTIMA^®^ 5 MS Accent fused-silica capillary columns (30 m × 0.25 mm × 0.25 μm; MACHEREY–NAGEL, Düren, GER). Helium (> 99.999%) was used as the carrier gas and passed through the column at a constant flow rate of 1 mL/min. The injection volume was 1 μL and the solvent delay time was 4.9 min. The temperature and energy of the electron impact ion were set to 230 °C and 70 eV, respectively. Data were obtained in full scan mode (m/z 50–600).

### Metabolomics data analysis

Following peak extraction, peak alignment, and deconvolution analysis in the automated mass spectral deconvolution and identification system (AMDIS, National Institute of Standards and Technology, USA), the raw data were transformed into a final data file containing observations (sample name), variables (rt_mz), and peak areas [28]. Additionally, the data were automatically matched against the mass spectral (MS) and retention time index (RI) (MSRI) libraries (version 2.0) freely available in the Golm Metabolome Database. The data were normalized and subjected to multivariate and univariate statistical analyses. For multivariate statistical analysis, orthogonal partial least squares-discriminant analysis (OPLS-DA) models were screened to elucidate the metabolic characterization among different clinical groups. Model quality was assessed by the R2Y and Q2 values, signifying goodness of fit and predictability, respectively. Differential metabolites were visualized in volcano plots. For univariate statistical analysis, Student's t-test was used to calculate the statistical significance of metabolites between two groups. The p < 0.05 from univariate statistical analysis and fold change (FC) > 1.2 were considered indicative of differential metabolites. Furthermore, a general linear regression analysis was performed to assess the effects of confounders on metabolites in different clinical subgroups. Pathway analysis of the differential metabolites was performed using MetaboAnalyst (Version 5.0).

### Statistical analysis

For descriptive analysis, differences in clinical variables were assessed using the Mann–Whitney U test for non-normally distributed variables and the chi-square test for categorical variables. Statistical significance was indicated as follows: *p < 0.05; **p < 0.01; ***p < 0.01. The Kruskal–Wallis’s test was used to compare variations in alpha diversity, including the Shannon, Simpson, and ACE indices. Additionally, Spearman rank correlation analysis was utilized to explore the potential association between intestinal genera and metabolites. Receiver Operating Characteristic curves (ROCs) were used to evaluate the predictive ability of unique microbial-metabolite features for disease states. Logistic regression was performed using forward and backward stepwise selection methods. All data were analyzed with Graph Pad Prism 8 software, R software (Version 2.15.3), and SPSS software (Version 21.0).

## Results

### Study cohort characteristics

A multi-omics approach was employed to investigate specific features of DCM subtypes through fecal microbial and metabolomics analysis. The final study cohort consisted of twenty-two patients with IDCM, twenty patients with ICM, and twenty HCs. Significant differences were observed in cardiac ultrasound parameters and underlying diseases between the HC and DCM (Table 1). As risk factors of coronary artery disease, hypertension (HTN) and type 2 diabetes mellitus (DM) were more prevalent in patients with ICM. RWMA and high Gensini scores were observed in ICM, contrasting with IDCM (Table 2). Importantly, patients in both the ICM and IDCM groups had a low proportion of previous medication use due to non-consultation or irregular drug administration.

**Table 1:** Clinical characteristics of HCs and DCM participants

Characteristics	HC (n = 20)	DCM (n = 42)	P
Age (year)	54.6	59.4	0.197
Sex (male)	16	33	0.897
Smoking	5	17	0.234
Alcohol intake	1	3	0.999
SBP (mmHg)	124	129	0.32
DBP (mmHg)	80	84	0.233
Diabetes mellitus	0	13	< 0.001
Hypertension	0	18	< 0.001
Atherosclerosis	0	4	< 0.001
LA (mm)	29.29	43.02	< 0.001
LVEDd (mm)	41.53	62.26	< 0.001
LVEDs (mm)	26.53	51.52	< 0.001
IVS (mm)	9.88	10.66	0.93
LVPW (mm)	9.65	10.45	0.04
RV (mm)	21.35	24.57	0.01
EF (%)	67.94	33.12	< 0.001
Cr (umol/L)	79.68	122.15	< 0.001
TC (mmol/L)	4.83	5.03	0.548
TG (mmol/L)	1.21	1.62	0.204
HDL (mmol/L)	1.41	1.08	0.001
LDL (mmol/L)	2.84	3.18	0.106

*SBP* systolic blood pressure, *DBP* diastolic blood pressure, *LA* left atrium, *LVEDd* left ventricular end-diastolic dimension, *LVEDs* left ventricular end-systolic dimension, *IVS* interventricular septum, *LVPW* left ventricular posterior wall, *RV* right ventricle, *EF* left ventricular ejection fraction, *Cr* creatinine, *TC* total cholesterol, *TG* triglyceride, *HDL* high density lipoprotein, *LDL* low density lipoprotein, *HC* healthy control, *DCM* dilated cardiomyopathy

**Table 2:** Clinical characteristics of ICM and IDCM participants

Characteristics	ICM (n = 20)	IDCM (n = 22)	P
Age (year)	63	56	0.043
Sex (male)	16	17	0.83
Smoking	9	8	0.654
Alcohol intake	1	2	0.607
SBP (mmHg)	127	131	0.644
DBP (mmHg)	81	87	0.165
Diabetes mellitus	13	0	< 0.001
Hypertension	13	5	0.006
Atherosclerosis	4	0	0.27
LA (mm)	40.7	45.14	0.071
LVEDd (mm)	57.75	66.36	< 0.001
LVEDs (mm)	47.05	55.59	< 0.001
IVS (mm)	10.94	10.41	0.313
LVPW (mm)	10.5	10.41	0.84
RV (mm)	23.5	25.55	0.143
EF (%)	34.65	31.73	0.178
RWMA	8	0	0.001
LDH (U/L)	255.68	255.68	0.687
HBD (U/L)	154.64	152.08	0.871
BUN (mmol/L)	9.79	7.77	0.084
Cr (umol/L)	144.08	102.23	0.013
TC (mmol/L)	4.94	5.11	0.776
TG (mmol/L)	1.67	1.57	0.801
HDL (mmol/L)	1.02	1.14	0.227
LDL (mmol/L)	3.15	3.20	0.891
Gensini scores	81.6	0.5	< 0.001
NT-proBNP(pg/ml)	6089.88	4277.78	0.423
CK-MB (ng/ml)	3.0017	4.23	0.419
MYO (ng/ml)	78.91	67.21	0.634
TnT (ng/ml)	136.14	105.75	0.707
Medication use			
ACEI/ARB	4	1	
CCB	3	2	
Biguanide hypoglycemic agents	7	0	
Sulphonylurea	5	0	
Statins	3	0	

*SBP* systolic blood pressure, *DBP* diastolic blood pressure, *HR* heart rate, *LA* left atrium, *LVEDd* left ventricular end-diastolic dimension, *LVEDs* left ventricular end-systolic dimension, *IVS* interventricular septum, *LVPW* left ventricular posterior wall, *RV* right ventricle, *LVEF* left ventricular ejection fraction, *RWMA* Regional wall motion abnormality, *LDH* lactate dehydrogenase, *HBD* hydroxyl butyric dehydrogenase, *BUN* blood urea nitrogen, *Cr* creatinine, *TC* total cholesterol, *TG* triglyceride, *HDL* high density lipoprotein, *LDL* low density lipoprotein, *CK-MB* creatine phosphokinase-isoenzyme-MB, *MYO* myoglobin, *TNT* troponin-T, *ICM* ischemic dilated cardiomyopathy, *IDCM* idiopathic dilated cardiomyopathy

### Gut microbial sequencing profiles

**Fig. 1:**
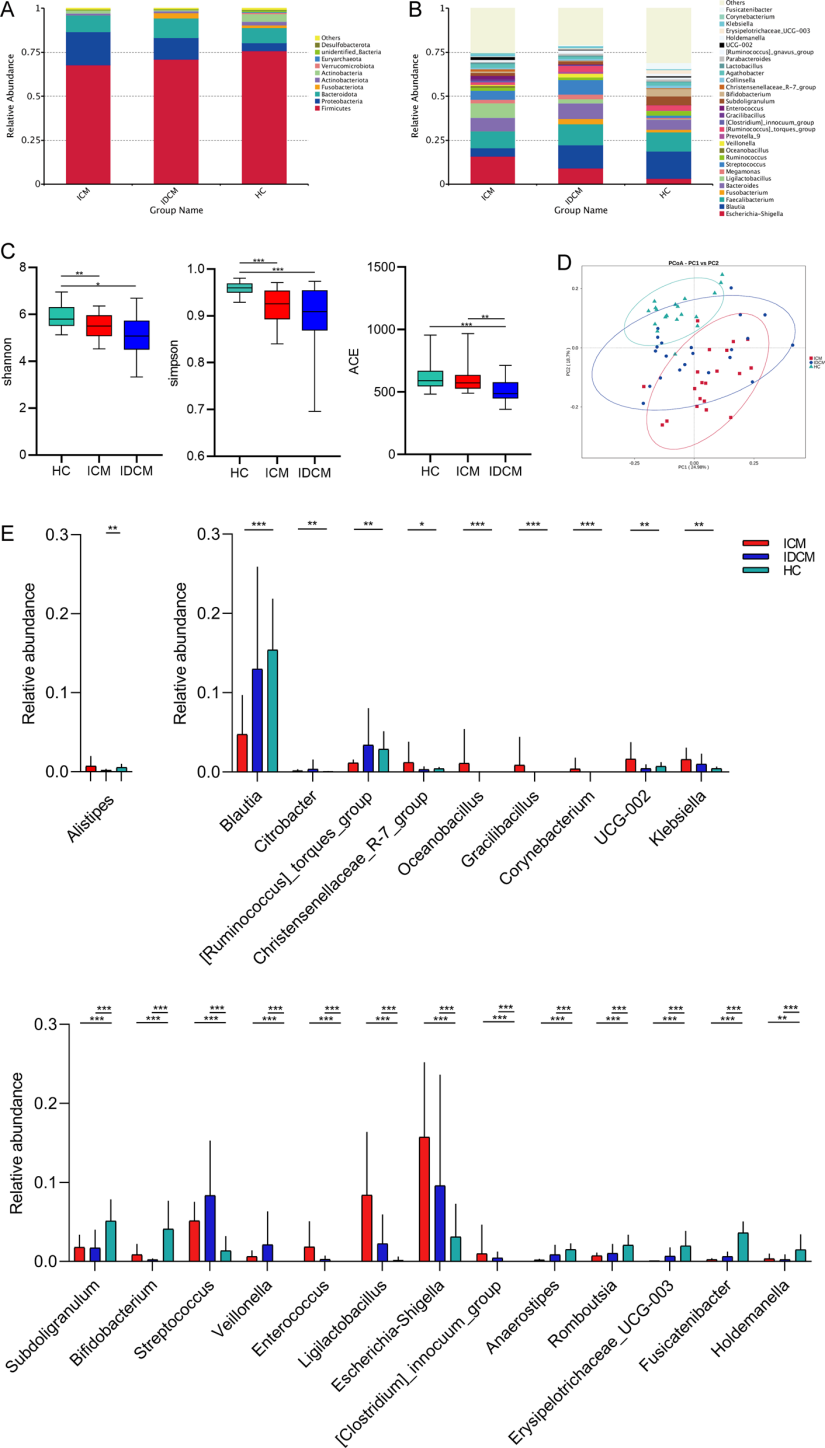
Alterations of the intestinal microbiota in disease groups and HC group. The relative abundances of **A** the top 10 phyla and **B** the top 30 genera clustered into ICM, IDCM, and HC. **C** Alpha diversity is measured by Shannon, Simpson and ACE index in ICM, IDCM and HC. Kruskal–Wallis’s test. **D** Principal coordinates analysis (PCoA) based on weighted-unifrac distances among ICM, IDCM, and HC. **E** At the genus level, box plots outline taxonomic features in ICM and IDCM compared to HC after adjusting for confounder. MaAsLin analysis. *P < 0.05, **P < 0.01, ***P < 0.001. *ICM* ischemic dilated cardiomyopathy, *IDCM* idiopathic dilated cardiomyopathy, *HC* health control

In total, approximately 4,092,873 effective tags were obtained and sorted into 2712 OTUs based on phylogenetic taxonomy. A comprehensive overview of taxonomic data was provided to describe the gut microbial structure across different clinical groups. At the phylum level, *Firmicutes*, *Proteobacteria*, *Bacteroidota* and *Actinobacteria* were the four main phyla in ICM and HC. In IDCM, *Firmicutes*, *Proteobacteria*, *Bacteroidota,* and *Fusobacteriota* were dominant, displaying clear differences compared to ICM and HC (Fig. 1A). At the genus level, most of the top 30 genera belonged to the *Firmicutes Phylum* in both ICM and IDCM. The total relative abundances of the top 30 genus accounted for 74.98% ± 6.23%, 79.18% ± 8.00%, and 69.18% ± 5.59% (Mean ± SD) in ICM, IDCM, and HC respectively (Fig. 1B, Additional file 1 and Additional file 2). Interestingly, the genera *Oceanobacillus* and *Gracilibacillus* were detected in both ICM and IDCM, but not in HC (Fig. 1B, Additional file 2).

### Gut microbiota signatures in participants with DCM subtypes and HCs

To investigate the gut microbial signatures associated with DCM subtypes, we assessed the diversity and composition of the gut microbiota in our study cohort. We observed a decrease in alpha diversity measured by the Shannon and Simpson indices in both DCM subtypes compared to HC (Shannon index: ICM vs HC, p < 0.01 and IDCM vs HC, p < 0.05; Simpson index: ICM vs HC, p < 0.001 and IDCM vs HC, p < 0.001; Kruskal–Wallis’s test) (Fig. 1C). Although there were no statistical differences in the Shannon and Simpson indices between the two groups, the ACE index was higher in ICM than in IDCM (ACE index: ICM vs IDCM, p < 0.01; Kruskal–Wallis’s test) (Fig. 1C). The PCoA based on weighted-unifrac distances was conducted to evaluate the overall microbial structures in individuals with cardiomyopathy and HC. The PCoA plot revealed distinct taxonomic components in ICM, IDCM, and HC (Fig. 1D). These findings indicate the presence of dysbiosis in the intestinal microbiota in different cardiomyopathy subtypes.

To explore the specific taxonomic features associated with DCM subtypes, we examined the relative abundances of different taxa among ICM, IDCM, and HC. We identified distinct components at various taxonomic levels, including 5 phyla, 7 families, and 22 genera, which exhibited differential abundances between ICM and HC (p < 0.05 and p < 0.01, Metastats analysis) (Additional file 3). Likewise, the relative abundances of 5 phyla, 4 families, and 17 genera in IDCM were significantly different from those in HC. (p < 0.05 and p < 0.01, Metastats analysis) (Additional file 3). At the genus level, MaAsLin was applied to control for potential confounders, including antihypertensive medications, antidiabetic agents, and statins. After correcting for confounding factors, 22 genera from ICM and 14 genera from IDCM retained statistical significance at the genus level (Fig. 1E, Additional file 3). Compared with HC, we further screened out taxonomic features that significantly changed in one DCM subtype (p < 0.05, MaAsLin analysis) but not in the other (p > 0.05, MaAsLin analysis) at the genus level. As a result, a characteristic combination of nine genera taxa including *Blautia, [Ruminococcus]_torques_group, Christensenellaceae_R-7_group, UCG-002, Corynebacterium, Oceanobacillus, Gracilibacillus, Klebsiella* and *Citrobacter* was specific to ICM. Only the genus *Alistipes* was found to be distinct in IDCM. Additionally, alterations in 13 genera taxa were observed in both ICM and IDCM compared to HC, indicating a considerable overlap of fecal microbiota in different cardiomyopathy subtypes (Fig. 1E).

### Fecal metabolic features in ICM and IDCM

**Fig. 2:**
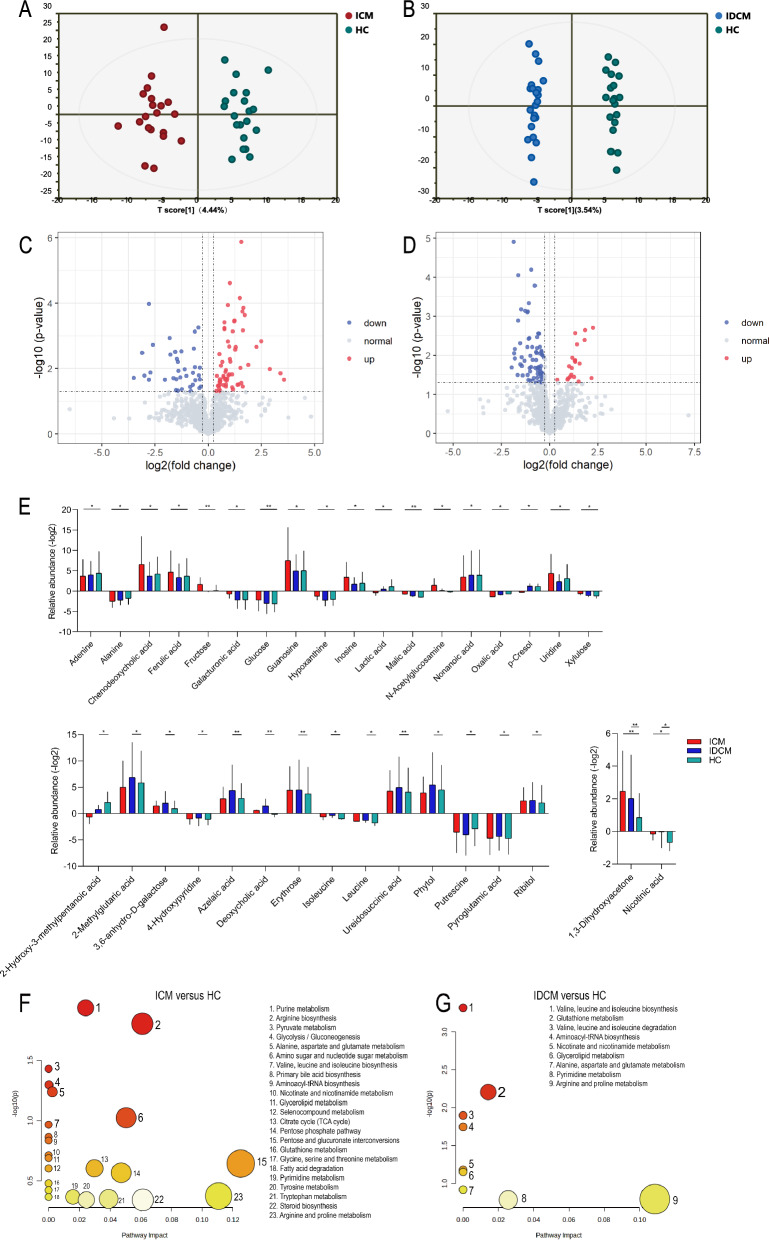
Overall pattern of fecal metabolome in ICM and IDCM. **A**, **B** The scatter plots of OPLS-DA show an obvious metabolic separation of ICM and IDCM compared to HC, respectively. Volcano plots reveal the metabolite alterations in **C** ICM and **D** IDCM versus HC. Up and down represented significantly elevated and decreased fecal metabolites in the disease group compared to HC. **E** Box plots show specific and common metabolites in ICM and IDCM after adjustment for confounders. *P < 0.05, **P < 0.01, ***P < 0.001. **F**, **G** Metabolome view of dysregulated pathways observed in ICM and IDCM compared with HC, respectively. *OPLS-DA* orthogonal partial least squares-discriminant analysis

To investigate metabolic alterations in different cardiomyopathy subtypes, we employed GC–MS analysis to assess fecal metabolites in ICM (n = 20), IDCM (n = 22), and HC (n = 20). A total of 70 annotated metabolites were identified in the fecal sample profiles. We utilized OPLS-DA to visualize the sample variability and identify possible outlier points between the groups. The OPLS-DA score plots revealed distinct patterns of metabolites between the clinical groups (ICM versus HC, R^2^Y = 0.928, Q^2^ = 0.481; IDCM versus HC, R^2^Y = 0.991, Q^2^ = 0.383) (Fig. 2A, B). Volcano plots further depicted the upregulation and downregulation of metabolites in ICM and IDCM. In comparison to HC, we identified 11 upregulated and 24 downregulated differential metabolites in ICM, whereas 15 reduced and 3 increased differential metabolites were found in IDCM (Fig. 2C, D, Additional file 4). Compared to HC, 20 metabolites in ICM and 16 metabolites in IDCM were significantly different after adjustment for confounders by a general linear regression analysis. Specifically, only two metabolites (1,3-dihydroxyacetone and nicotinic acid) were common alterations in ICM and IDCM. (Fig. 2E, Additional file 4).

Pathway analysis using KEGG annotation was conducted to investigate the metabolic characteristics of ICM and IDCM. Three metabolic pathways, namely purine metabolism, arginine biosynthesis and pyruvate metabolism were closely associated with ICM. In addition, disturbances in valine, leucine and isoleucine biosynthesis were found in ICM (Fig. 2F, G).

### Stratification for the association of intestinal genus and metabolites with DCM according to DM and HTN status

**Fig. 3:**
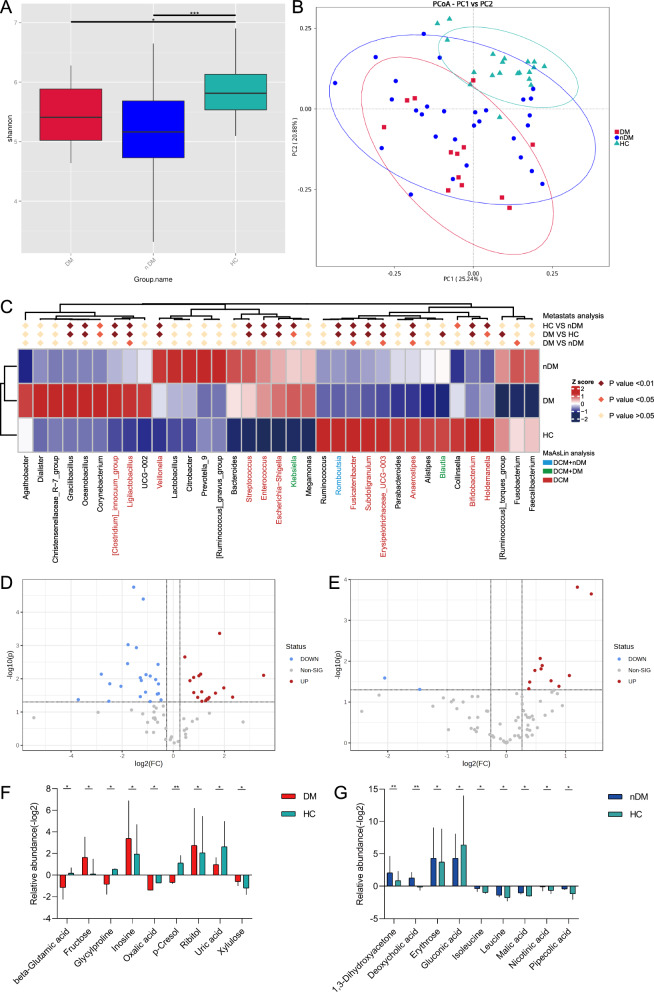
Stratification of the intestinal microbial and metabolic signatures in DCM according to DM status. **A** Alpha diversity measured by Shannon index. Kruskal–Wallis’s test. **B** PCoA depicts bacterial composition among DCM-DM, DCM-nDM and HC. **C** Heatmap outlines taxonomic features in DCM subgroups at genus level by Metastats analysis and after adjustment for confounder by MaAsLin analysis, respectively. Volcano plots revealed the metabolite alterations in **D** DCM-DM and **E** DCM-nDM versus HC. Box plots show metabolites altered in **F** DCM-DM and **G** DCM-nDM compared to HC, adjusted for confounding factors. *P < 0.05, **P < 0.01, ***P < 0.001. *DCM-DM* dilated cardiomyopathy with diabetes mellitus, *DCM-nDM* dilated cardiomyopathy without diabetes mellitus

Stratified analyses were applied to investigate the relevance of intestinal genus and metabolites in patients with DCM according to DM and HTN status. Patients with DCM were separated into DM (DCM-DM, n = 13) and non-DM (DCM-nDM n = 29) subgroups. Compared to HC, Shannon indices were significantly lower in both DCM subgroups (DCM-DM vs HC, p < 0.05 and DCM-nDM vs HC, p < 0.001; Kruskal–Wallis’s test) (Fig. 3A). PCoA plot based on weighted-unifrac distances visualized heterogeneity of gut microbiota among DCM-DM, DCM-nDM, and HC (Fig. 3B). At the genus level, the relative abundances of 19 genera in DCM-DM and 18 genera in DCM-nDM were significantly different compared to HC (Fig. 3C). After correcting for confounders, two genus including *Blautia* and *Klebsiella* was found distinctly in DCM-DM, while only one genus *Romboutsia* was specific to DCM-nDM. In addition, we found 12 genera belonging to DCM with and without DM (Fig. 3C). Meanwhile, faecal untargeted metabolomics data were stratified according to DM status. Compared with HC, 40 and 13 differential metabolites were detected in DCM-DM and DCM-nDM patients, respectively (Fig. 3D, E). Possibly influenced by drugs, we found only nine specific metabolites for either DCM-DM or DCM-nDM after adjustment for confounding factors including antihypertensive medications, antidiabetic agents, and statins (Fig. 3F, G).

**Fig. 4:**
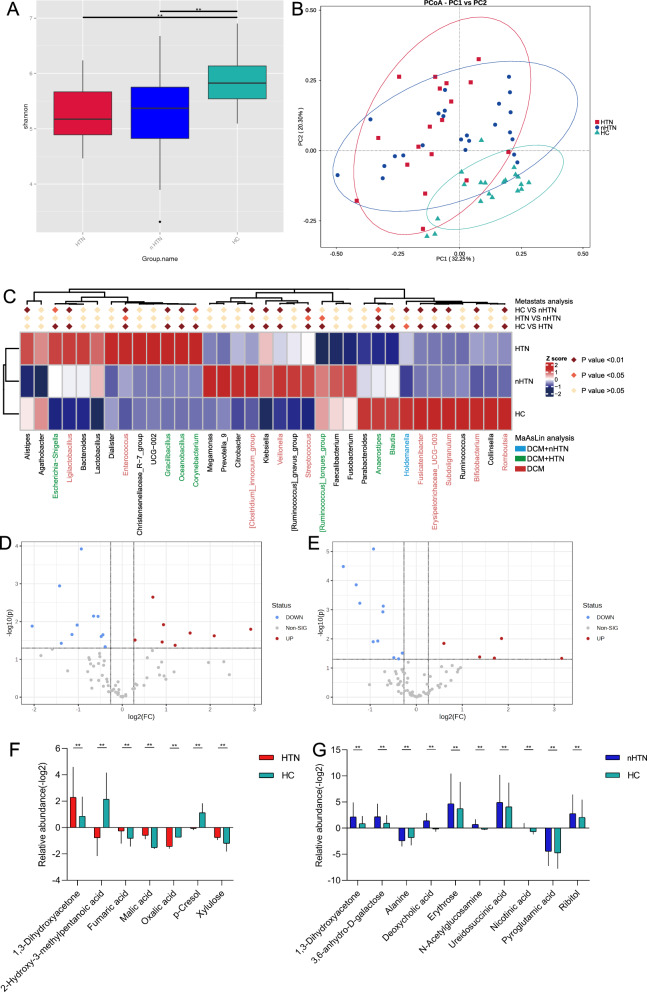
Stratification of the intestinal microbial and metabolic characteristics in DCM according to HTN status. **A** Alpha diversity measured by Shannon index in DCM-HTN, DCM-nHTN and HC. Kruskal–Wallis’s test. **B** PCoA based on weighted-unifrac distances among different clinical groups. **C** Heatmap outlines taxonomic features in DCM-HTN and DCM-nHTN at genus level. Volcano plots reveal the metabolite alterations in **D** DCM-HTN and **E** DCM-nHTN versus HC. Box plots show the relative abundance of metabolites in DCM-HTN **F** and DCM-nHTN **G** compared to HC, adjusted for confounding factors. *P < 0.05, **P < 0.01, ***P < 0.001. *DCM-HTN* dilated cardiomyopathy with hypertension, *DCM-nHTN* dilated cardiomyopathy without hypertension

Likewise, we further classified patients with DCM into two subgroups based on HTN status (DCM with HTN, DCM-HTN, n = 18 and DCM with non-HTN, DCM-nHTN, n = 24). Consistently, Kruskal–Wallis’s test depicted a lower Shannon index in the DCM-HTN and DCM-nHTN group than in the HC group (Fig. 4A). PCoA illustrated that microbiota communities in individuals with DCM-HTN and DCM-nHTN significantly differed from HCs (Fig. 4B). To identify the microbiological profile associated with HTN, the compositions of the gut microbiota were explored using Metastat analysis. At the genus level, this analysis revealed 19 and 18 different abundant taxa for DCM-HTN and DCM-nHTN compared to HC, respectively (Fig. 4C). After adjusting for confounders, the relative abundances of *Escherichia-Shigella, Gracilibacillus, Oceanobacillus,* and *Corynebacterium* were enriched in DCM-HTN patients, whereas the relative abundances of *[Ruminococcus]_torques_group, Anaerostipes* and *Blautia* were enriched in HC. We found a decrease in the relative abundance of *Holdemanella* in DCM-nHTN patients compared to HC. In addition, 10 genera significantly altered in both DCM subgroups compared to the HC group (Fig. 4C). As mentioned above, the changes in fecal metabolites were also identified in DCM subgroups correlated with HTN status. Currently, 19 and 16 significantly altered metabolites were identified in DCM-HTN and DCM-nHTN groups, as shown in volcano diagrams (Fig. 4D, E). After correction for confounders, one metabolite was significantly higher in both DCM groups than in HC. 6 metabolites in DCM-HTN and 9 metabolites in DCM-nHTN were markedly distinct from those in HC (Fig. 4F, G).

### Correlation analysis between fecal genus and metabolites

**Fig. 5:**
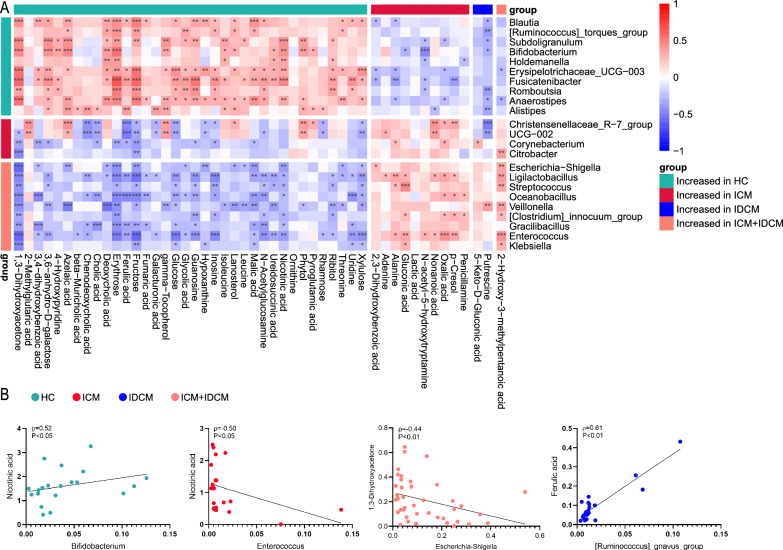
**A** Heatmap showed the potential links between differential genus and metabolites. Spearman rank correlation analysis, *P < 0.05, **P < 0.01, ***P < 0.001. **B** Examples of genus and metabolite associations

To explore the correlation between fecal genera and metabolites, a correlation analysis was performed using altered genera and metabolites in clinical groups. A heatmap was generated to visualize the potential associations between differential genera and metabolites (Fig. 5A, Additional file 5 and Additional file 6). These results demonstrated positive or negative correlations between specific bacterial genera and metabolite parameters under different pathophysiological conditions. For example, the level of nicotinic acid exhibited a positive association with enriched genera such as *Bifidobacterium* and *Holdemanella* in HC, while it showed a negative association with *Escherichia-Shigella* and *Enterococcus* in ICM (Fig. 5A, B). The genera *Escherichia-Shigella*, which increased in ICM, displayed a negative correlation with 1,3-dihydroxyacetone (Fig. 5B). Although there are likely numerous mechanistic associations between bacteria and metabolites, further experiments are necessary to determine their causal relationship.

### Discriminating disease status based on multi-omics analysis

**Fig. 6:**
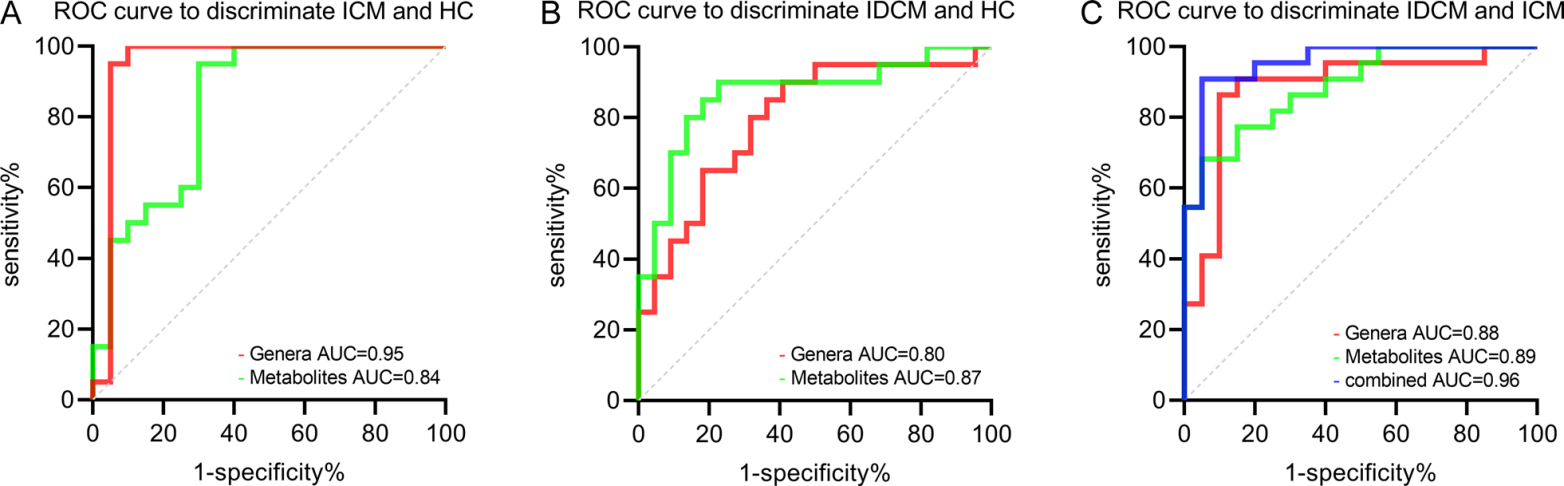
ROC curves analysis to verify the predictive value of microbial and metabolic features in **A** ICM and HC, **B** IDCM and HC, and **C** ICM and IDCM

To further verify the effectiveness of the specific genera and derived metabolites in disease prediction, we selected seven genera (*Blautia, [Ruminococcus]_torques_group, Christensenellaceae_R-7_group, UCG-002, Corynebacterium, Citrobacter,* and *Alistipes*) and seven metabolites (2-methylglutaric acid, azelaic acid, chenodeoxycholic acid, ferulic acid, deoxycholic acid, galacturonic acid and pyroglutamic acid) to distinguish disease status. Using a microbial signature composed of the seven genera, it was possible to identify ICM from HC with an AUC value of 0.95. The combination of the seven metabolites also showed a favorable diagnostic value with an AUC of 0.84 (Fig. 6A). The microbial (AUC = 0.80) and metabolic signatures (AUC = 0.87) were effective in discriminating IDCM from HC, respectively. (Fig. 6B). Furthermore, the gut microbial genus (AUC = 0.88) and metabolite model (AUC = 0.89) were comparable in predicting IDCM from ICM. Especially, the combination of fecal microbial-metabolic features improved the ability to differentiate IDCM from ICM, achieving an AUC of 0.96 (Fig. 6C). Overall, the integration of intestinal microbial and metabolite features holds promise for predicting disease status in clinical practice.

## Discussion

Cardiomyopathy encompasses a range of complex syndromes characterized by organ dysfunction. Identifying the differential etiologies of cardiomyopathy poses a substantial challenge in clinical practice. In this study, we employed a multi-omics approach to explore the gut microbiota and fecal metabolomics characteristics of patients with ICM and IDCM. Commonalities observed between ICM and IDCM include reduced alpha diversity and alterations in microbial structure as evidenced by beta diversity. Our findings are consistent with prior studies. For instance, Luedde et al. [29] reported decreased Shannon diversity indices in ICM and IDCM patients when compared to HC. This study highlighted significant differences in the overall microbiological composition between DCM patients and HC. Similar results emerged from our OPLS-DA score plots, suggesting distinctive metabolite profiles across DCM subtypes and HC. These findings collectively underscore structural and functional disruptions within the gut microbiota in both ICM and IDCM. Importantly, we profiled distinct genera and metabolites in the faeces of patients with ICM and IDCM compared to HC. We also demonstrated the promising potential of microbial-metabolite features in distinguishing between ICM and IDCM.

Interestingly, we identified unique genera and metabolites in the feces of ICM and IDCM compared to HC. Specific taxonomy variations emerged between ICM, IDCM, and HC at the phylum level. *Firmicutes*, *Proteobacteria*, *Bacteroidota* and *Actinobacteria* were the four main phyla in ICM and HC. While *Firmicutes*, *Proteobacteria*, *Bacteroidota,* and *Fusobacteriota* were dominant in IDCM. Notably, *Actinobacteria* exhibited lower abundance in ICM patients compared to HC, consistent with prior research [30, 31]. In contrast, the *Proteobacteria* phylum was notably increased in both DCM groups, which is noteworthy due to its potential role in immune dysfunction, inflammatory diseases, and metabolic disorders [32, 33]. Amar et al. [34] reported an elevation in circulating *Proteobacteria* as a distinct indicator of cardiovascular events, thereby emphasizing its potential relevance as an independent marker in the context of cardiovascular health. Furthermore, findings by Koren et al. [35] revealed a notable abundance of *proteobacteria* within coronary atherosclerotic plaques, implying a potential proinflammatory role of *Proteobacteria* in the processes underlying plaque formation within the coronary vasculature. Previous investigations have highlighted several genera within *Proteobacteria* phylum, including *Haemophilus*, *Cardiobacterium,* and *Eckenella corrosiveum*, has potential contributors to diseases such as endocarditis, vasculitis, and myocarditis [36, 37]. At the genus level, a collection of nine specific taxa namely *Blautia, [Ruminococcus]_torques_group, Christensenellaceae_R-7_group, UCG-002, Corynebacterium, Oceanobacillus, Gracilibacillus, Klebsiella,* and *Citrobacter* were distinctive to ICM, whereas the taxon *Alistipes* exhibited unique alterations in IDCM after accounting for confounding factors. Of note, we observed *Streptococcus*, known as a microbiological marker of ischemic heart disease severity [17, 38], to be notably enriched in both ICM and IDCM patients relative to HC. These findings highlighted the potential significance of *streptococcus* in differentiating disease states from HC. Consequently, comprehensive cross-sectional studies are imperative to unravel the interplay between cardiomyopathy and gut microbiota. The genus *Citrobacter* is a unique group of human pathogens involved in emotional disorders, drug resistance, and opportunistic infections. For example, the abundance of *Citrobacter werkmanii* was associated with neuroactive metabolites such as kynurenic acid and gamma-aminobutyric acid in patients with depression [39]. *Citrobacter rodentium* released inflammatory damage-associated molecular patterns that induced endothelial dysfunction and the formation of thrombosis [40]. We found a distinct increase of *Citrobactor werkmanii* in ICM compared to HC after confounder adjustments. Interestingly, some strains of the genus *Citrobacter* may have anti-angiosclerotic properties. Notably, the microbial enzyme trimethylamine lyase catalyzes the conversion of phosphatidylcholine and choline to produce trimethylamine within the gut lumen, which is subsequently transported to the liver and metabolized into trimethylamine-N-oxide, a putative promoter of atherosclerosis [41]. *Citrobacter amalonaticus,* isolated from human faecal samples, demonstrated the capacity to anaerobically utilise choline, thereby inhibiting trimethylamine accumulation [42]. These studies suggest a potentially significant role of *Citrobacter amalonaticus* in an alternative pathway for chronic degradation, possibly inhibiting plaque formation. Interestingly, the environmental bacteria *Oceanobacillus* and *Gracilibacillus* were detected in the DCM and found to be associated with HTN states after adjusting for confounders. This genus was isolated from high-salt environments such as salt fields and fermented food [43, 44]. Huang et al. [45] found that several strains of *Gracilibacillus* produce indole acetic acid, a product of the gut microbiota in uremic patients. Previous studies revealed indole acetic acid-induced cardiac injury in patients with end-stage renal disease, including cardiac hypertrophy and diastolic dysfunction [46]. Furthermore, serum indole acetic acid was a significant predictor of mortality and cardiovascular events in chronic kidney disease patients [47]. The role of *Gracilibacillus* in DCM requires further study. Building upon specific genera, our endeavors led to the development of a non-invasive microbiological marker to differentiate between ICM, IDCM, and HC. The amalgamation of seven unique genera yielded substantial discrimination between ICM from HC, achieving an AUC value of 0.95. While the differentiation of IDCM from HC exhibited relatively diminished performance with an AUC of 0.80, possibly attributed to the scarcity of distinct genera in IDCM, novel models are warranted to enhance the predictive accuracy for IDCM.

The gut microflora achieves extensive interactions with the host by metabolic exchange. We further analysed the relationship between distinct faecal metabolites and the occurrence of ICM, and IDCM. We observed 18 unique metabolites in the ICM, mainly including carbohydrates, purines and purine nucleosides (e.g., guanosine, hypoxanthine, and inosine), carbohydrate conjugates (e.g., fructose, galacturonic acid, and xylulose), some hydroxy acids and pyrimidine nucleosides. In our study, the relative abundance of hypoxanthine was lower in ICM than in HC. When hypoxanthine is converted to uric acid by xanthine oxidase, superoxide is produced, which is toxic to cardiomyocytes [48]. Previous studies have shown that xanthine oxidase in the myocardium increased activity in the presence of atherosclerosis [49]. Inhibition of xanthine oxidase reduces superoxide accumulation and improves myocardial contractility in patients with ICM [48, 50]. Tang et al. [51] found that malic acid significantly reduced myocardial infarct size and cardiomyocyte apoptosis in a model of myocardial ischaemia–reperfusion injury. Uridine, a pyrimidine nucleoside, was decreased in ICM. Krylova et al. [52] revealed that uridine prevents myocardial injury by activating mitochondrial ATP-dependent potassium channels in both ischaemic and ischaemia/reperfusion modes. In addition, there are 14 specific metabolites in IDCM, dominated by amino acids (e.g., isoleucine, leucine, ureidosuccinic acid and pyroglutamic acid), carbohydrates (e.g., erythrose and ribitol) and fatty acids (e.g., 2-methylglutaric acid and azelaic acid). Gong et al. [53] performed a study of serum metabolomic analysis to predict response to cardiac resynchronisation therapy in patients with IDCM. They found that isoleucine and linoleic acid were potential biomarkers in distinguishing responders from non-responders. In mouse models of myocarditis and dilated cardiomyopathy infected with coxsackievirus B3, circulating leucine and isoleucine are diagnostic biomarkers for chronic myocarditis and DCM, respectively [54]. We believe that further studies are necessary to investigate the potential role of amino acids and other metabolites in IDCM. The combination of seven metabolic features (2-methylglutaric acid, azelaic acid, chenodeoxycholic acid, ferulic acid, deoxycholic acid, galacturonic acid, and pyroglutamic acid) was able to distinguish ICM (AUC = 0.84) and IDCM (AUC = 0.87) from HC. This metabolite model separated IDCM from ICM with an AUC of 0.89. Furthermore, the combination of fecal microbial-metabolic features was performed accurately to differentiate IDCM from ICM with an AUC of 0.96.

We applied stratified analyses to investigate the influence of comorbidities, including DM and HTN on the specific genus and metabolites. *Klebsiella* is a potential human pathogen associated with multidrug resistance, infections in immunocompromised individuals and multiple metabolic disorders [55]. Previous clinical studies have shown a strong link between the increased abundance of *Klebsiella* and the presence and severity of coronary artery disease [18, 56]. Another study found diverse bacteria such as *Klebsiella* colonization in the atherosclerotic plaques of patients with coronary artery disease [57]. In our study, *Klebsiella* was more enriched in ICM than HC after adjusting for confounding factors. Further stratification revealed that the relative abundance of *Klebsiella* was significantly impacted by diabetic status. An increased proportion of *Klebsiella* has been reported in patients with obesity and DM [17]. On the other hand, several genera including *Klebsiella* have been shown to serve as a microbial marker for the diagnosis of diabetic nephropathy [58]. Based on these findings, improving intestinal dysbiosis may be a promising strategy for treating DM and its complications. Ferulic acid, a natural phenol found in lignocellulose, is recognized for its biological activity in anti-inflammatory, anti-tumor, and cardiovascular protection. Prior studies have shown that ferulic acid attenuates the migratory and proliferative capacity of cardiac fibroblasts and reduces myocardial fibrosis after myocardial infarction by inhibiting the pRB-E2F1/CCNE2 and RhoA/ROCK2 pathways [59]. Moreover, ferulic acid protects cardiomyocytes and mouse hearts from severe endoplasmic reticulum stress through the activation of the PERK/eIF2α/ATF4/CHOP pathway [60]. Although ferulic acid has been previously reported to have benefits in DM and HTN, its relative abundance in ICM was not influenced by disease stratification in our study [61, 62]. Therefore, the microbial and metabolite features implicated in the pathophysiological process of myocardial disease and comorbidities require further investigation.

Moreover, our analysis indicated that both DCM subtypes shared common microbial taxa and metabolites compared to the HC group. Nicotinic acid, a precursor of nicotinamide adenine dinucleotide (NAD) and nicotinamide adenine dinucleotide phosphate, is an important regulator of cardiac energy metabolism [63, 64]. We found that nicotinic acid consistently decreased in ICM and IDCM. Previous studies have reported that ischemia in cardiomyocytes resulted in NAD + depletion, reduced available ATP, and mitochondrial dysfunction [65]. Supplementation of nicotinic acid restored NAD + balance, improved abnormal blood flow conditions and alleviated ischemia–reperfusion injury [66]. Similarly, NAD + levels showed a significant decrease in different types of DCM [67, 68]. In a virus-induced mouse model, metabolic analysis of cardiac tissue revealed that nicotinate and nicotinamide metabolism was involved in the progression of DCM [69]. Additionally, we observed a positive correlation between nicotinic acid levels and the abundance of several potential probiotics in HC, while it was negatively correlated with pathogenic genera in both DCM subtypes. This suggests that intestinal microbial dysbiosis may disrupt the balance of energy metabolism in cardiomyopathies. The genus *Bifidobacterium*, belonging to the phylum *Actinobacteria*, consists of dozens of Gram-positive, polymorphic rod-shaped subspecies resident in the gastrointestinal tract of humans and mammals. *Bifidobacterium* has the ability to inhibit the growth of harmful bacteria, improve intestinal barrier function, and reduce pro-inflammatory cytokines. Recent research has also shown that *Bifidobacterium* alters the function of dendritic cells to maintain the immune balance of the intestine and initiate protection against pathogens [70, 71]. Supporting our results, the abundance of *Bifidobacterium* was lower in patients with coronary artery disease than in HCs [72]. Furthermore, oral administration of probiotics containing *Bifidobacterium* has been shown to relieve depression and anxiety in patients with coronary artery disease [73]. Probably, several alterations in genus-metabolite signatures are concomitant signs of diverse diseases, indicating potential diagnostic roles in disease states [70, 74].

Several limitations exist in this cross-sectional study. Firstly, the low prevalence of ICM and IDCM resulted in a relatively small sample size in the clinical cohort, highlighting the need for multicenter data to validate the results. Secondly, due to the study design, we were unable to observe the dynamic changes in gut microbiota and metabolites during the development of cardiomyopathies. A longitudinal study may provide insights into the alterations of fecal microbiota and metabolites before and after the onset of cardiomyopathy. Furthermore, although our data revealed a link between fecal microbiota, derived metabolites, and disease phenotypes, we did not provide information on their causal relationships. Further functional and molecular studies are warranted to explore the potential mechanisms underlying these diseases.

In summary, this study has unveiled significant alterations in the composition and functional perturbations of the intestinal microbiota across distinct types of cardiomyopathies, shedding light on their potential contributions to the underlying pathogenesis of these conditions. The intricate and multifaceted interplay between the intestinal microbiota and metabolites further underscores their pivotal role in the context of cardiomyopathy. Notably, specific microbial-metabolite features have shown promising diagnostic utility in discriminating between various forms of cardiomyopathies. These findings offer novel insights into the potential etiological factors influencing cardiomyopathy and illuminate the substantial involvement of the intestinal microbiota in the evolution of these cardiac disorders. Further investigations delving into the mechanistic links and therapeutic implications of the observed microbial and metabolic alterations could pave the way for innovative strategies aimed at the management and treatment of cardiomyopathies.

### Supplementary Information

**Additional file 1. **The microbial relative abundances in patients with ICM, IDCM and HCs at pylum level.

**Additional file 2. **The microbial relative abundances in patients with ICM, IDCM and HCs at genus level.

**Additional file 3. **Comparative analysis of taxonomic features at the phylum (table 1), family (table 2), and genus (table 3) levels in ICM and IDCM, respectively.

**Additional file 4. **Nontargeted metabolomics analysis of ICM, IDCM and HC by GC–MS.

**Additional file 5. **Taxa_metabolite.spearman.pMatrix.

**Additional file 6. **Taxa_metabolite.spearman.corMatrix.

## Data Availability

All data generated or analysed during this study are included in this published article.

## Abbreviations

DCM: Dilated cardiomyopathy
ICM: Ischemic dilated cardiomyopathy
IDCM: Idiopathic dilated cardiomyopathy
HC: Healthy control
HTN: Hypertension
DM: Diabetes mellitus
DCM-DM: Dilated cardiomyopathy with diabetes mellitus
DCM-nDM: Dilated cardiomyopathy without diabetes mellitus
DCM-HTN: Dilated cardiomyopathy with hypertension
DCM-nHTN: Dilated cardiomyopathy without hypertension
OTU: Operational taxonomic unit
RWMA: Regional wall motion abnormality
PCoA: Principal coordinate analysis
OPLS-DA: Orthogonal partial least squares-discriminant analysis
GC–MS: Gas chromatography–mass spectrometry
ROC: Receiver operating characteristic
AUC: Area under curve
SBP: Systolic blood pressure
DBP: Diastolic blood pressure
LA: Left atrium
LVEDd: Left ventricular end-diastolic dimension
LVEDs: Left ventricular end-systolic dimension
IVS: Interventricular septum
LVPW: Left ventricular posterior wall
RV: Right ventricle
LVEF: Left ventricular ejection fraction
Cr: Creatinine
TC: Total cholesterol
TG: Triglyceride
HDL: High density lipoprotein
LDL: Low density lipoprotein
CK-MB: Creatine phosphokinase-isoenzyme-MB
MYO: Myoglobin
TNT: Troponin-T
NAD: Nicotinamide adenine dinucleotide
